# Supplementary material for: P-TRAP: a Panicle Trait Phenotyping tool
Source: BMC Plant Biol. 2013 Aug 29;13:122. doi: 10.1186/1471-2229-13-122 (PMC3848748; doi:10.1186/1471-2229-13-122)
Supplement: Additional file 7 — P-TRAP source code. The Netbeans project that contains the source code of the application. [file 1471-2229-13-122-S7.zip › javadoc.zip!/javadoc/org-bioalg-riceprojecttype/index-all.html]

Index (RiceProjectType)


JavaScript is disabled on your browser.


- Package
- Class
- Tree
- Deprecated
- Index
- Help

*org.bioalg.riceprojecttype 1.0*

- Prev
- Next

- Frames
- No Frames

- All Classes

G I L O P R S 


## G

getLookup() - Method in class org.bioalg.riceprojecttype.RiceProject


GetParticleResultFolder(boolean) - Method in class org.bioalg.riceprojecttype.RiceProject


getProjectDirectory() - Method in class org.bioalg.riceprojecttype.RiceProject


getRiceFolder(boolean) - Method in class org.bioalg.riceprojecttype.RiceProject


getRiceGrainResultsMasterFile(boolean) - Method in class org.bioalg.riceprojecttype.RiceProject


getRiceImageFolder(boolean) - Method in class org.bioalg.riceprojecttype.RiceProject


getRiceResultsDetailsFile(boolean) - Method in class org.bioalg.riceprojecttype.RiceProject


getRiceResultsFolder(boolean) - Method in class org.bioalg.riceprojecttype.RiceProject


getRiceResultsImageFolder(boolean) - Method in class org.bioalg.riceprojecttype.RiceProject


getRiceResultsMasterFile(boolean) - Method in class org.bioalg.riceprojecttype.RiceProject


## I

IMAGE\_DIR - Static variable in class org.bioalg.riceprojecttype.RiceProjectFactory


isProject(FileObject) - Method in class org.bioalg.riceprojecttype.RiceProjectFactory


## L

loadProject(FileObject, ProjectState) - Method in class org.bioalg.riceprojecttype.RiceProjectFactory


## O

org.bioalg.riceprojecttype - package org.bioalg.riceprojecttype


## P

PARTICLES\_RESULT\_DIR - Static variable in class org.bioalg.riceprojecttype.RiceProjectFactory


PARTICLES\_RESULT\_FILE - Static variable in class org.bioalg.riceprojecttype.RiceProjectFactory


PROJECT\_DIR - Static variable in class org.bioalg.riceprojecttype.RiceProjectFactory


## R

RESULTS\_DETAILS\_FILE - Static variable in class org.bioalg.riceprojecttype.RiceProjectFactory


RESULTS\_DIR - Static variable in class org.bioalg.riceprojecttype.RiceProjectFactory


RESULTS\_MASTER\_FILE - Static variable in class org.bioalg.riceprojecttype.RiceProjectFactory


RiceProject - Class in org.bioalg.riceprojecttype


RiceProject(FileObject, ProjectState) - Constructor for class org.bioalg.riceprojecttype.RiceProject


RiceProjectFactory - Class in org.bioalg.riceprojecttype
:   The projects creator class.

RiceProjectFactory() - Constructor for class org.bioalg.riceprojecttype.RiceProjectFactory


## S

saveProject(Project) - Method in class org.bioalg.riceprojecttype.RiceProjectFactory

G I L O P R S

- Package
- Class
- Tree
- Deprecated
- Index
- Help

*org.bioalg.riceprojecttype 1.0*

- Prev
- Next

- Frames
- No Frames

- All Classes
